# Efficacy and Safety of Infliximab and Vedolizumab Maintenance Therapy in Patients with Crohn’s Disease and Ulcerative Colitis: A Systematic Review and Meta-Analysis

**DOI:** 10.3390/jcm14134419

**Published:** 2025-06-21

**Authors:** Bruce E. Sands, Laurent Peyrin-Biroulet, Stefan Schreiber, Silvio Danese, Alessandro Armuzzi, Anthony Buisson, Mathurin Fumery, Axel Dignass, Nick Powell, Nicholas A. Kennedy, Sebastian Zeissig, Taek Kwon, Seungmin Kim, Kyoungwan Nam, Stephen B. Hanauer

**Affiliations:** 1 Dr. Henry D. Janowitz Division of Gastroenterology, Icahn School of Medicine at Mount Sinai, New York, NY 10029, USA; 2 Department of Hepato-Gastroenterology and INSERM U954 NGERE, University Hospital of Nancy, University of Lorraine, 54500 Nancy, France; 3 Paris IBD Center, Groupe Hospitalier Privé Ambroise Paré-Hartmann, 92200 Neuilly-sur-Seine, France; 4 Department of Medicine I, University Hospital Schleswig-Holstein, 24105 Kiel, Germany; 5 Department of Gastroenterology, IRCCS San Raffaele Hospital, 20132 Milan, Italy; 6 IBD Center, IRCCS Humanitas Research Hospital, Rozzano, 20089 Milan, Italy; 7 Department of Biomedical Sciences, Humanitas University, 20072 Milan, Italy; 8 Department of Hepato-Gastroenterology, University Hospital Estaing of Clermont-Ferrand, 63100 Clermont-Ferrand, France; 9 Department of Hepatogastroenterology, Université Clermont Auvergne, 63000 Clermont-Ferrand, France; 10 CHU Amiens, Université de Picardie Jules Verne, Unité Péritox, 80054 Amiens, France; 11 Department of Medicine I, Agaplesion Markus Hospital, Goethe University Frankfurt, 60431 Frankfurt am Main, Germany; axel.dignass@agaplesion.de; 12 Department of Metabolism, Digestion and Reproduction, Faculty of Medicine, Imperial College London, London W12 0NN, UK; 13 Royal Devon University Healthcare NHS Foundation Trust, Exeter EX2 5DW, UK; 14 Center for Regenerative Therapies Dresden, Technische Universität Dresden, 01307 Dresden, Germany; 15 Department of Internal Medicine A, University Medicine Greifswald, 17475 Greifswald, Germany; 16 Celltrion, Inc., Incheon 22014, Republic of Korea; 17 Division of Gastroenterology and Hepatology, Department of Medicine, Feinberg School of Medicine, Northwestern University, Chicago, IL 60611, USA

**Keywords:** clinical trials, endoscopy, inflammatory bowel disease, infliximab, vedolizumab

## Abstract

**Background/Objectives:** Direct comparative data for infliximab and vedolizumab are limited due to lack of head-to-head trials. This systematic review and meta-analysis compared the efficacy and safety of infliximab and vedolizumab as intravenous or subcutaneous maintenance treatments for adults with moderately to severely active Crohn’s disease or ulcerative colitis. **Methods:** Medical databases, PubMed, Embase, and the Cochrane Library were systematically searched from January 2010 to May 2024 to identify Phase 1 to 3 randomized controlled trials. The primary and co-primary outcomes were the proportions of patients achieving clinical remission and clinical response at one year, respectively. Safety was also analyzed (PROSPERO CRD42023483599). Data for each outcome were pooled using a two-sided random-effects model in separate analyses for Crohn’s disease and ulcerative colitis. **Results:** Seven eligible Crohn’s disease trials and eight eligible ulcerative colitis trials contributed data for 1910 and 2372 patients, respectively. For Crohn’s disease, higher proportions of infliximab-treated patients achieved clinical remission (0.64 [95% confidence interval: 0.60–0.68]) and/or clinical response (0.71 [0.67–0.75]) at one year compared with vedolizumab-treated patients (0.40 [0.35–0.46] and 0.47 [0.43–0.51], respectively). For ulcerative colitis, similar proportions of infliximab- and vedolizumab-treated patients achieved clinical remission (0.54 [0.38–0.71] vs. 0.40 [0.35–0.44]) and/or clinical response (0.52 [0.45–0.58] vs. 0.58 [0.51–0.65]) at one year. Safety results showed no significant differences. **Conclusions:** An indirect comparison of maintenance treatment with infliximab and vedolizumab demonstrated that infliximab yields significantly better efficacy than vedolizumab in Crohn’s disease, whereas both agents yielded similar efficacy in ulcerative colitis.

## 1. Introduction

The tumor necrosis factor inhibitor (TNFi) infliximab (IFX) and the α4/β7 integrin blocker vedolizumab (VDZ) are recommended first-line biologic treatments for patients with moderately to severely active inflammatory bowel disease (IBD) [1,2]. In patients with Crohn’s disease (CD), IFX is recommended to induce remission in those who have not responded to conventional therapy, while VDZ is recommended to induce remission in those with an inadequate response to conventional therapy and/or to anti-TNF therapy [2]. The same agent that has been used to induce clinical response is recommended for subsequent maintenance of clinical remission [2]. In patients with ulcerative colitis (UC), IFX and VDZ are each recommended to induce remission in individuals with an inadequate response or intolerance to conventional therapy, and the same agent used to induce remission is recommended for subsequent maintenance treatment [1]. A previous systematic review and meta-analysis indirectly comparing IFX and VDZ reported better efficacy with IFX compared with VDZ as induction treatment in patients with CD or UC [3]. Furthermore, a recent comparative analysis of IFX and VDZ reported significantly better efficacy for intravenous (IV) IFX versus VDZ as induction therapy [4]. Given the chronic, relapsing nature of IBD and the need for sustained disease control, a comparative evaluation of IFX and VDZ in the maintenance phase is both clinically relevant and necessary to inform long-term treatment strategies. Therefore, comparison of IFX and VDZ as maintenance therapy is warranted.

IFX and VDZ have been available as IV formulations for IBD for many years, while subcutaneous (SC) formulations have been approved more recently [5,6,7,8]. CT-P13 SC is the first SC formulation of IFX to gain approval in Europe and the USA for IBD maintenance treatment [6,7]. Several large-scale, Phase 3, randomized, placebo-controlled trials in patients with IBD were conducted to support US regulatory approval of IFX SC (LIBERTY-CD; LIBERTY-UC) [9] and VDZ SC (VISIBLE 1, VISIBLE 2) [10,11].

In the absence of head-to-head trial data for IFX and VDZ, several systematic reviews and meta-analyses have compared the efficacy and safety of these agents [12,13,14]. Peyrin-Biroulet and colleagues conducted a network meta-analysis of various maintenance regimens of IFX and VDZ, demonstrating that IFX SC ranked highest for the achievement of clinical remission compared with other IFX IV and VDZ IV/SC regimens [4,15]. In addition, a recent comparative analysis reported a numerically higher achievement of clinical remission with maintenance IFX IV than with VDZ in patients with moderate-to-severe CD, and similar efficacy between IFX SC and VDZ maintenance therapy for patients with moderate-to-severe UC [4]. To build on this work, given the availability of important new data from the LIBERTY-CD/UC [9] and VISIBLE 2 trials [10], which have studied SC formulations of the respective substances, we conducted an updated systematic literature review and meta-analysis to compare one-year efficacy and safety data for IFX (IV or SC) and VDZ (IV or SC) in patients with moderately to severely active CD and UC. This study adds valuable updated evidence on the comparative efficacy and safety of IFX and VDZ, incorporating new data on SC formulations to better inform clinical practice.

This systematic literature review and meta-analysis was conducted according to a prospectively registered study protocol (PROSPERO number CRD42023483599) [16].

## 2. Search Strategy

We conducted systematic electronic searches of PubMed, Embase, and the Cochrane Library, and manual searches of abstracts from major gastroenterology conferences (i.e., Digestive Diseases Week, European Crohn’s and Colitis Organisation Congress, United European Gastroenterology Week); core search terms were developed using Medical Subject Headings and free-text terms and adapted for each database (e.g., Crohn’s disease, ulcerative colitis, infliximab, vedolizumab, randomized controlled trial; Table S1). All searches were performed for the period of 1 January 2010 through 22 May 2024 and were restricted to English-language publications reporting human studies. Reference lists from relevant systematic reviews were cross-checked against the electronic search results.

## 3. Inclusion and Exclusion Criteria

### 3.1. Study Design

Clinical studies covering Phases 1 to 3 were eligible for inclusion, but these were required to be parallel-group randomized (placebo- or active-) controlled trials. Protocols (without results), case reports/studies, notes, commentaries, letters, editorials, opinions, and economic model studies were excluded.

### 3.2. Participants

Two cohorts of patients were included and analyzed separately: adults (aged ≥ 18 years) with moderately to severely active CD or adults with moderately to severely active UC. Pediatric patients were excluded.

### 3.3. Interventions

The prespecified intervention of interest was IFX (IV or SC) as maintenance treatment, and the prespecified comparator of interest was VDZ (IV or SC) as maintenance treatment. Patients receiving maintenance treatment were included from both treat-through study designs and studies involving re-randomization of responders.

## 4. Outcome Measures

Reporting of one or more of the following prespecified outcomes of interest in the trial was not an inclusion criterion for the review.

### 4.1. Primary Efficacy Outcomes

The primary outcome was the proportion of patients who achieved clinical remission at 1 year (weeks 52–54; patients with CD: an absolute Crohn’s Disease Activity Index [CDAI] score ≤ 150. Patients with UC: a total/partial Mayo score ≤ 2 with no individual sub-score >1; or a partial Mayo score ≤ 1 with a rectal bleeding sub-score of 0; or a modified Mayo score with a stool frequency sub-score of ≤1, rectal bleeding sub-score of 0, and endoscopic sub-score of ≤1).

The co-primary outcome was the proportion of patients who achieved clinical response at 1 year (weeks 52–54; a decrease in the CDAI score of ≥100 points from the baseline value in patients with CD or a decrease in the total Mayo score from a baseline of ≥3 points and ≥30% [or a decrease in the partial Mayo score from a baseline of ≥2 points and ≥25%], with an accompanying decrease in the rectal bleeding sub-score of ≥1 or an absolute rectal bleeding sub-score of ≤1, in patients with UC).

### 4.2. Secondary Efficacy Outcomes

Secondary efficacy outcomes were the proportion of patients achieving mucosal healing (e.g., an absence of mucosal abnormalities [i.e., Simple Endoscopic Score-CD score ≤ 2] in patients with CD or a Mayo endoscopic sub-score ≤ 1 in patients with UC) and the proportion of patients achieving corticosteroid (CS)-free remission (e.g., clinical remission at week 52/54 without CS therapy [patients with CD] among patients using CS at baseline).

### 4.3. Safety Outcomes

Safety outcomes were the proportions of patients experiencing adverse events (AEs), serious adverse events (SAEs), serious infections, and AEs leading to discontinuation.

## 5. Evidence Synthesis and Quality Assessment

### 5.1. Study Selection

Two authors (S.M.K., K.N.) independently screened the titles and abstracts of the retrieved records to identify studies as either potentially relevant for inclusion or as irrelevant to the research question and to be excluded (noting reasons). A third reviewer (T.S.K.) considered a randomly chosen sample of excluded studies to verify that the exclusion criteria were applied correctly.

Full-text publications for possibly relevant studies were obtained and independently reviewed by two authors (S.M.K., K.N.) to establish inclusion/exclusion. Where necessary, disagreements were settled by discussion/arbitration by a third author (T.S.K.). Multiple reports of the same study were collated, making studies the unit of interest. A Preferred Reporting Items for Systematic Reviews and Meta-Analyses (PRISMA) flow chart was used to document the study selection process [17].

### 5.2. Data Extraction and Management

Study/participant characteristics and outcome data were extracted from eligible studies by two authors (S.M.K., K.N.) and checked by a third author (T.S.K.). Extracted data were recorded using a Microsoft Excel template (Microsoft Corp., Redmond, WA, USA).

### 5.3. Quality Assessment

Risks of bias for the included studies were assessed using the Cochrane Risk of Bias tool (version 1.0) [18]. Briefly, the following domains were judged as being at a high, low, or unclear risk of bias: random sequence generation; allocation concealment; blinding of participants and personnel; blinding of outcome assessment; incomplete outcome data; selective outcome reporting; and other bias. Quality assessments were performed by two authors responsible for data extraction (S.M.K., K.N.) and checked by a third author (T.S.K.).

## 6. Data Synthesis and Measures of Treatment Effect

Data for each prespecified outcome were pooled in two separate analyses for patients with CD and UC. Sensitivity analyses were conducted to examine the effect of the exclusion of data for patients receiving IFX IV or VDZ IV (i.e., to examine treatment effects with SC formulations), the exclusion of data for TNFi-experienced patients (i.e., to examine treatment effects in TNFi-naïve individuals), and the exclusion of treat-through study designs (i.e., to examine treatment effects in trials involving re-randomization of responders).

Data from multiple studies were pooled using a two-sided random-effects model to account for within-study and between-study variability. For each outcome, pooled data were reported as forest plots with 95% confidence intervals (CIs). Effect sizes and 95% CIs were compared between IFX and VDZ, and a statistically significant difference in effect size between IFX and VDZ was inferred if the respective 95% CI did not overlap [19]. Heterogeneity in treatment effects across trials was estimated using the *I*^2^ statistic, to quantify the proportion of total variation in study estimates due to heterogeneity rather than chance. Statistical analyses were performed using the MetaProp function in R (version 4.2.2; R Foundation, Vienna, Austria). The pooled estimates of effect size were computed, and heterogeneity was evaluated and reported accordingly.

## 7. Results

### Search Results

**Figure 1:**
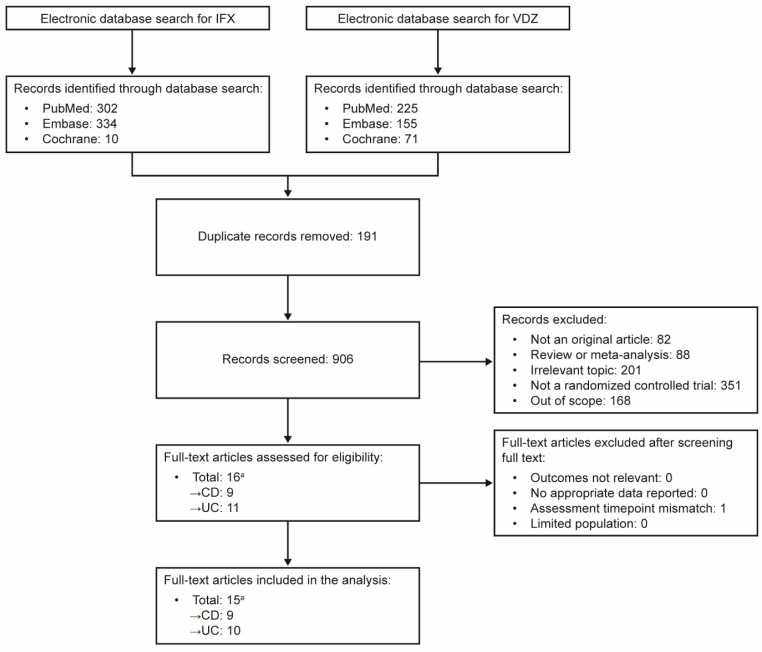
PRISMA flow diagram. ^a^ Four articles, describing the CT-P13 1.6 [23], LIBERTY-CD and -UC [9], and NOR-SWITCH [20,21] studies, included results in patients with IBD (CD, UC). CD, Crohn’s disease; IBD, inflammatory bowel disease; IFX, infliximab; PRISMA, Preferred Reporting Items for Systematic Reviews and Meta-analyses; UC, ulcerative colitis; VDZ, vedolizumab.

A PRISMA flow diagram summarizing the flow of information in this systematic review is presented in Figure 1. Briefly, we identified 1097 records through electronic and hand searches. After the removal of duplicates, 906 records were screened (890 articles excluded) and 16 full-text articles were assessed against the eligibility criteria; 15 articles, reporting seven studies in patients with CD and seven studies in patients with UC, were included in the qualitative synthesis and quantitative analyses.

In patients with CD, five studies were identified for IFX and included in the quantitative synthesis (SONIC [20], NOR-SWITCH [21,22], PLANET-CD [23], NCT02883452 [CT-P13 SC 1.6] [24], NCT03945019 [LIBERTY-CD] [9]), and two studies were identified for VDZ and included in the quantitative synthesis (NCT00783692 [GEMINI 2] [25,26], NCT02611817 [VISIBLE 2] [10]).

In patients with UC, four studies were identified for IFX and included in the quantitative synthesis (ACT 1 [27,28], NOR-SWITCH [21,22], NCT02883452 [CT-P13 SC 1.6] [24], NCT04205643 [LIBERTY-UC] [9]), and three studies were identified for VDZ and included in the quantitative synthesis (NCT00783718 [GEMINI 1] [29,30], NCT02497469 [VARSITY] [31], NCT02611830 [VISIBLE 1] [11]). Despite the primary analysis of ACT 1 being published earlier than the search window, it was included in a post hoc analysis published in 2022 [28] and was thus included in the present study.

## 8. Study Characteristics

### 8.1. Crohn’s Disease

A summary of study designs and participant characteristics for the included studies for CD is presented in Table S2. All seven studies were randomized trials with a duration of 50–54 weeks; six included a double-blind maintenance period and one employed an open-label design (CT-P13 SC 1.6). Three studies were placebo controlled (LIBERTY-CD, GEMINI 2, VISIBLE 2) while four employed active controls (CT-P13 SC 1.6, NOR-SWITCH, PLANET-CD, SONIC). Only one study (SONIC) evaluated combination therapy in a randomized treatment arm (azathioprine plus IFX IV 5 mg/kg every 8 weeks [Q8W]). The PLANET-CD, NOR-SWITCH, and CT-P13 SC 1.6 studies included switching phases.

IV formulations of IFX or VDZ were evaluated in ≥1 arm of five of the seven studies; the doses evaluated were IFX 5 mg/kg Q8W, VDZ 300 mg every four weeks (Q4W), or VDZ 300 mg Q8W. SC IFX was evaluated in the CT-P13 SC 1.6 study (120/240 mg every two weeks [Q2W]) and in the LIBERTY-CD study (120 mg Q2W; patients who had lost their initial clinical response by week 22 were escalated to 240 mg Q2W and considered non-responders/non-remitters in the present analysis); SC VDZ (108 mg Q2W) was evaluated in the VISIBLE 2 study.

Data from 1019 patients receiving IFX and 891 patients receiving VDZ contributed to the quantitative analyses for CD. Where reported at baseline, the mean/median age ranged from 29.0 to 39.5 years, 44.2 to 61.8% of participants were male, and the mean/median disease duration ranged from 2.2 to 14.3 years. The mean/median body weight was reported for the SONIC, GEMINI 2, and VISIBLE 2 studies and ranged from 68.5 to 74.1 kg, while the median BMI was reported for the CT-P13 SC 1.6 and LIBERTY-CD studies and ranged from 21.8 to 23.6 kg/m^2^.

### 8.2. Ulcerative Colitis

A summary of study designs and participant characteristics for the included studies for UC is presented in Table S3. All seven studies were randomized trials with a duration of 52–54 weeks; six included a double-blind maintenance period and one study (CT-P13 SC 1.6) employed an open-label design. Four studies were placebo-controlled (ACT 1, LIBERTY-UC, GEMINI 1, VISIBLE 1) while three employed active controls (CT-P13 SC 1.6, NOR-SWITCH, VARSITY). The NOR-SWITCH and CT-P13 SC 1.6 studies included switching phases in their design.

All studies enrolling patients with UC included ≥1 maintenance treatment arm to evaluate IFX or VDZ as monotherapy. IV formulations of IFX or VDZ were evaluated in ≥1 arm of six of the seven studies; the doses evaluated were IFX 5 mg/kg Q8W, IFX 10 mg/kg Q8W, VDZ 300 mg Q4W, or VDZ 300 mg Q8W. SC IFX was evaluated in the CT-P13 SC 1.6 study (120/240 mg Q2W) and in the LIBERTY-UC study (120 mg Q2W; patients who had lost their initial clinical response by week 22 were escalated to 240 mg Q2W and considered non-responders/non-remitters in the present analysis). SC VDZ (108 mg Q2W) was evaluated in the VISIBLE 1 study.

Data from 1056 patients receiving IFX and 1316 patients receiving VDZ contributed to the quantitative analyses for UC. Where reported at baseline, the mean/median age ranged from 33.0 to 45.8 years, 53.8 to 69.6% of participants were male, and the mean/median disease duration ranged from 5.7 to 11.5 years. The mean body weight was reported for the ACT 1, GEMINI 1, VARSITY, and VISIBLE 1 studies and ranged from 71.6 to 80.0 kg, while the median BMI was reported for the CT-P13 SC 1.6 and LIBERTY-UC studies and ranged from 23.3 to 24.7 kg/m^2^.

## 9. Risk of Bias in the Included Studies

A summary of the risk of bias assessment is presented in Figure S1. Across the 84 assessments performed (12 studies and 7 risk of bias domains), 75 were considered to be at low risk of bias and 9 were considered at high risk of bias. The GEMINI 1, GEMINI 2, VISIBLE 1, VISIBLE 2, LIBERTY-UC, LIBERTY-CD, and PLANET-CD studies were all ‘randomized-responder’ study designs and thus considered to be at high risk of ‘other bias’ due to the selective inclusion of induction responders in the maintenance phase. The CT-P13 SC 1.6 study was judged to be at high risk of performance bias, due to an open-label design.

## 10. Comparative Efficacy and Safety Between Treatments

### 10.1. Efficacy Analyses

**Figure 2:**
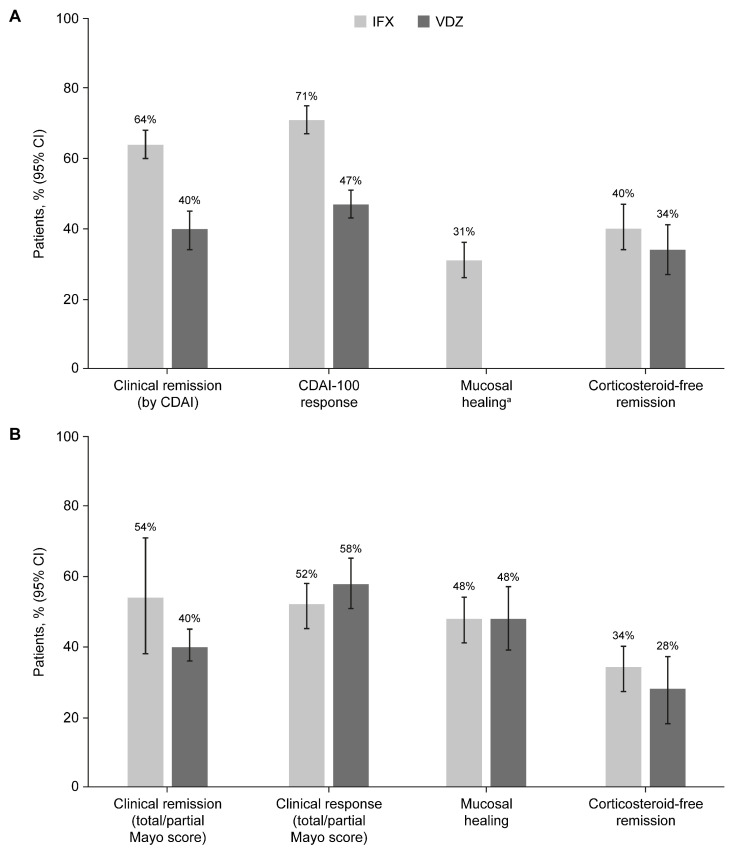
Pooled rates (95% CI) of efficacy outcomes during maintenance treatment with IFX or VDZ in patients with CD (**A**) and UC (**B**). ^a^ No mucosal healing data were available for patients with CD treated with VDZ. CD, Crohn’s disease; CDAI, Crohn’s Disease Activity Index; CI, confidence interval; IFX, infliximab; UC, ulcerative colitis; VDZ, vedolizumab.

**Figure 3:**
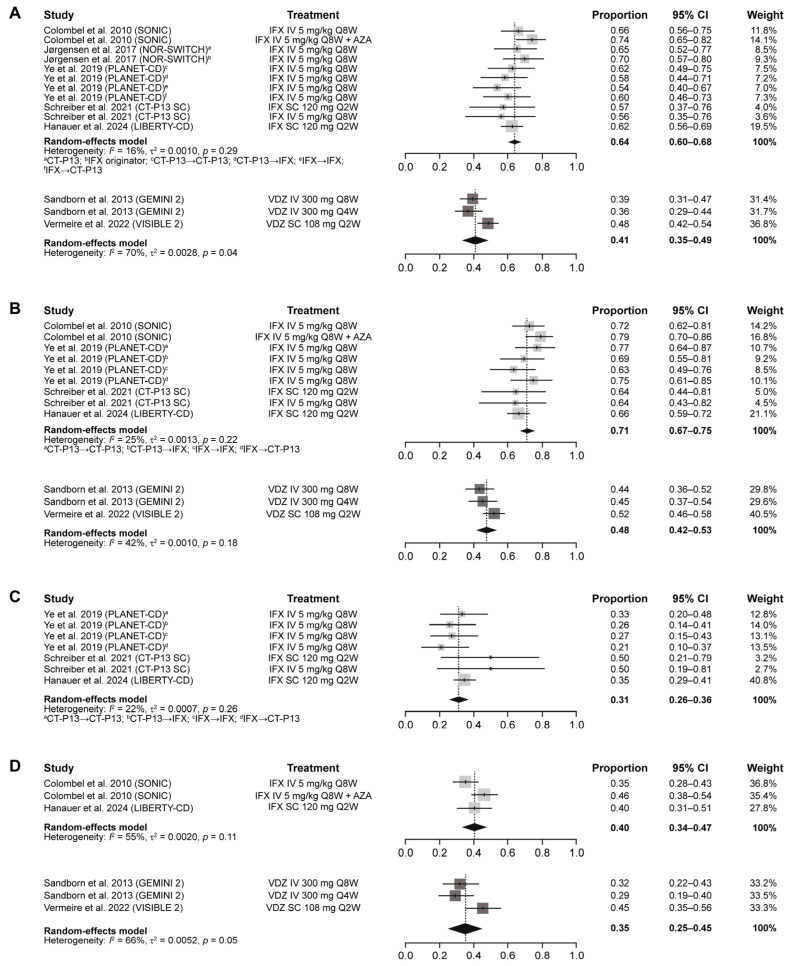
Pooled rates of clinical remission by CDAI (**A**), clinical response by CDAI-100 (**B**), mucosal healing or endoscopic remission (**C**), and corticosteroid-free remission (**D**) during maintenance treatment with IFX or VDZ in patients with CD [9,10,20,21,23,24,25]. Note: due to rounding, some totals are not 100%. CD, Crohn’s disease; CDAI, Crohn’s Disease Activity Index; CI, confidence interval; IFX, infliximab; IV, intravenous; Q*n*W, every *n* weeks; SC, subcutaneous; UC, ulcerative colitis; VDZ, vedolizumab.

The results of the meta-analyses for the comparison between IFX and VDZ in patients with CD are presented in Table 1. Pooled results for the primary and co-primary efficacy outcomes in patients with CD showed that a higher proportion of patients who received IFX maintenance treatment achieved clinical remission and clinical response compared with patients who received VDZ maintenance treatment, with non-overlapping 95% CIs indicating statistical significance (Figure 2A) [32]. Specifically, the pooled rates of clinical remission by CDAI score throughout the one-year maintenance period were 0.64 (95% CI: 0.60–0.68) with IFX and 0.41 (95% CI: 0.35–0.49) with VDZ (Figure 3A), and the pooled rates of clinical response by CDAI-100 throughout the one-year maintenance period were 0.71 (95% CI: 0.67–0.75) with IFX and 0.48 (95% CI: 0.42–0.53) with VDZ (Figure 3B). In terms of secondary efficacy outcomes in patients with CD, the pooled rate of mucosal healing throughout the one-year maintenance period with IFX was 0.31 (95% CI: 0.26–0.36) (Figure 3C), and there were no mucosal healing data for VDZ in patients with CD. The pooled rates of CS-free remission throughout the one-year maintenance period were numerically higher with IFX (0.40 [95% CI: 0.34–0.47]) than with VDZ (0.35 [95% CI: 0.25–0.45]) (Figure 3D).

**Figure 4:**
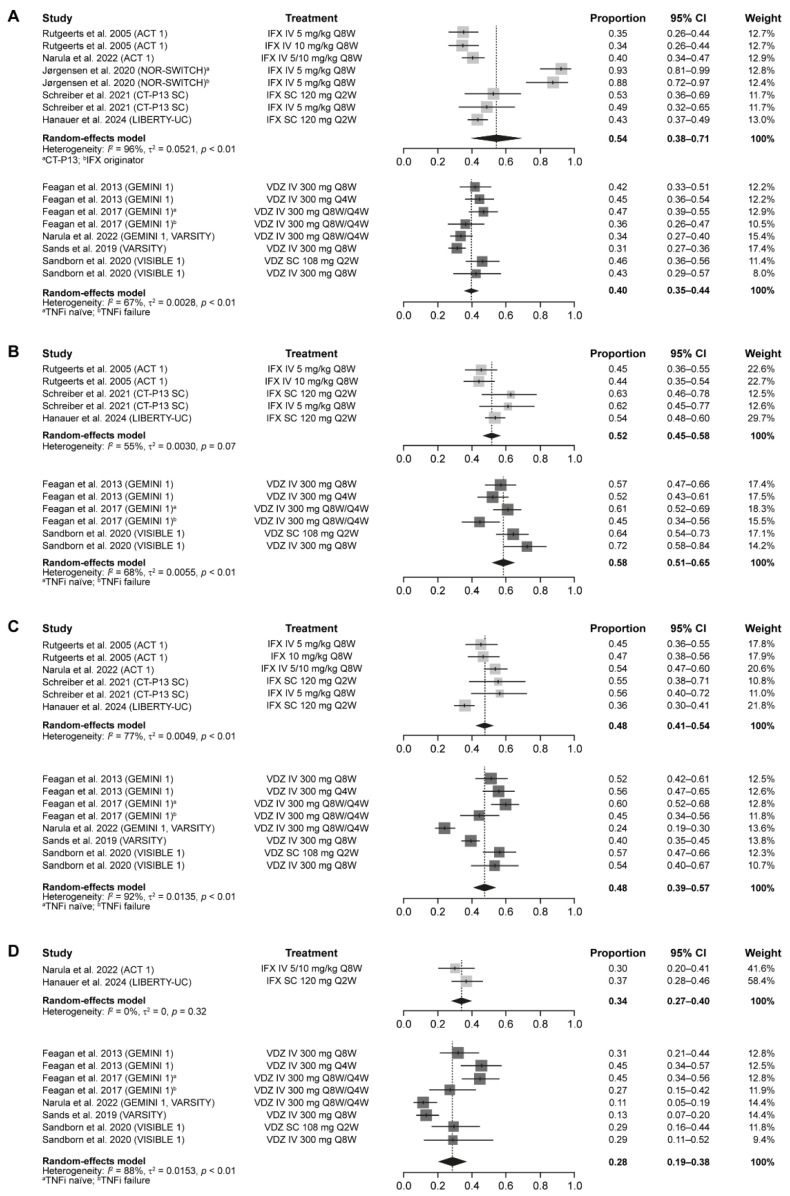
Pooled rates of clinical remission by total/partial Mayo score (**A**), clinical response by total/partial Mayo score (**B**), mucosal healing (**C**), and corticosteroid-free remission (**D**) during maintenance treatment with IFX or VDZ in patients with UC [9,11,22,24,27,28,29,30,31]. Note: due to rounding, some totals are not 100%. CI, confidence interval; IFX, infliximab; IV, intravenous; Q*n*W, every *n* weeks; SC, subcutaneous; TNFi, tumor necrosis factor inhibitor; UC, ulcerative colitis; VDZ, vedolizumab.

The results of the meta-analyses for the comparison between IFX and VDZ in patients with UC are presented in Table 2. The pooled results for the primary, co-primary, and secondary efficacy outcomes in patients with UC showed that similar proportions of patients achieved clinical remission and clinical response with IFX and VDZ maintenance treatment, with the overlapping 95% CIs indicating that the differences were statistically non-significant (Figure 2B). Specifically, the pooled rates throughout the one-year maintenance period were as follows: 0.54 (95% CI: 0.38–0.71) with IFX and 0.40 (95% CI: 0.35–0.44) with VDZ for clinical remission by total/partial Mayo score (Figure 4A); 0.52 (95% CI: 0.45–0.58) with IFX and 0.58 (95% CI: 0.51–0.65) with VDZ for clinical response by total/partial Mayo score (Figure 4B); 0.48 (95% CI: 0.41–0.54) with IFX and 0.48 (95% CI: 0.39–0.57) with VDZ for mucosal healing (Figure 4C); and 0.34 (95% CI: 0.27–0.40) with IFX and 0.28 (95% CI: 0.19–0.38) with VDZ for CS-free remission (Figure 4D).

### 10.2. Sensitivity Analyses

The pooled results for the primary and co-primary efficacy outcomes in patients treated with IFX SC (Figure S2; no pooled data were available for VDZ SC) were consistent with those reported in the main analyses (i.e., SC or IV dosing). In patients with CD treated with IFX SC, the pooled rate of clinical remission by CDAI was 0.61 (95% CI: 0.56–0.67), and the pooled rate of clinical response by CDAI-100 was 0.65 (95% CI: 0.60–0.71). In patients with UC treated with IFX SC, the pooled rates of clinical remission by total/partial Mayo score and clinical response by total/partial Mayo score were 0.45 (95% CI: 0.40–0.50) and 0.56 (95% CI: 0.50–0.63), respectively.

The pooled results for the primary and co-primary efficacy outcomes in VDZ-treated, TNFi-naïve/-experienced patients with CD (Figure S3) or UC (Figure S4) were numerically higher than those reported in the main analyses (including VDZ-treated, TNFi-experienced patients), but the trend was generally consistent when comparing with IFX in the main analyses. In VDZ-treated, TNFi-naïve patients with CD, the pooled rate of clinical remission by CDAI was 0.49 (95% CI: 0.42–0.55), the pooled rate of clinical response by CDAI was 0.56 (95% CI: 0.50–0.62), and the pooled rate of CS-free remission was 0.42 (95% CI: 0.33–0.51). In TNFi-experienced patients with CD, the pooled rate of clinical remission by CDAI was 0.41 (95% CI: 0.26–0.56), the pooled rate of clinical response by CDAI was 0.37 (95% CI: 0.19–0.55), and the pooled rate of CS-free remission was 0.33 (95% CI: 0.07–0.58). In VDZ-treated, TNFi-naïve patients with UC, the pooled rate of clinical remission by total/partial Mayo score was 0.43 (95% CI: 0.34–0.51), the pooled rate of mucosal healing was 0.42 (95% CI: 0.22–0.62), and the pooled rate of CS-free remission was 0.35 (95% CI: 0.16–0.53).

Pooled results for the primary and co-primary efficacy outcomes in IFX-treated and VDZ-treated patients with CD in trials involving re-randomization of responders (Figure S5; no pooled data were available for UC-treated patients) were consistent with those reported in the main analyses (i.e., both treat-through study designs and those involving re-randomization of responders). In IFX-treated patients with CD, the pooled rate of clinical remission by CDAI was 0.61 (95% CI: 0.56–0.65), and the pooled rate of clinical response by CDAI-100 was 0.69 (95% CI: 0.64–0.74). In VDZ-treated patients with CD, the pooled rate of clinical remission by CDAI was 0.41 (95% CI: 0.34–0.49), and the pooled rate of clinical response by CDAI-100 was 0.48 (95% CI: 0.42–0.53).

### 10.3. Safety Analyses

**Figure 5:**
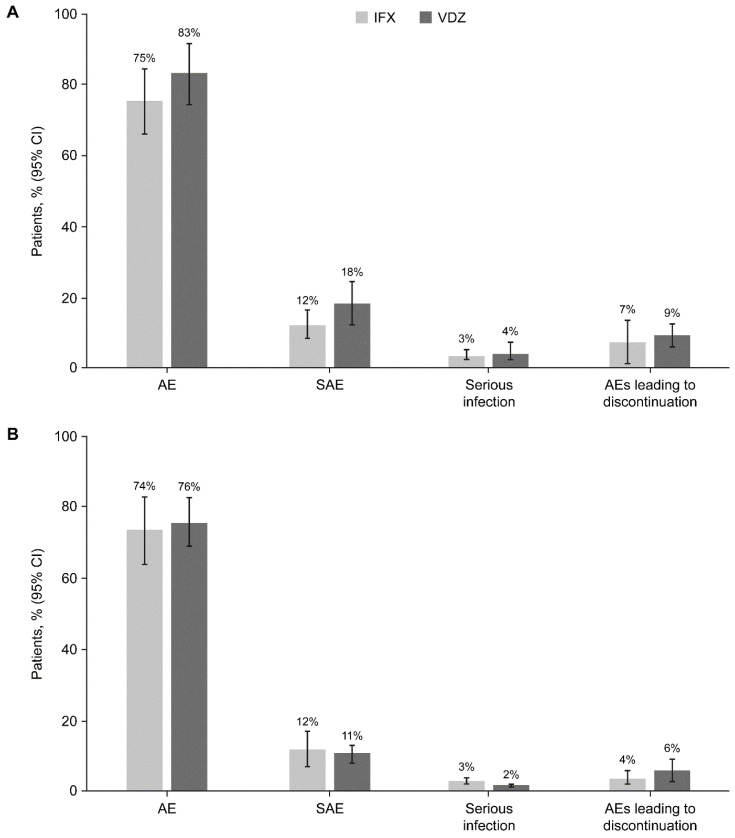
Pooled rates (95% CI) of AEs, SAEs, serious infection, and discontinuation due to AEs during maintenance treatment with IFX or VDZ in patients with CD (**A**) and UC (**B**). AE, adverse event; CD, Crohn’s disease; CI, confidence interval; IFX, infliximab; SAE, serious adverse event; UC, ulcerative colitis; VDZ, vedolizumab.

Pooled results for safety outcomes showed no significant differences in the proportions of patients who experienced AEs, SAEs, serious infections, or discontinuation due to AEs between treatment with IFX and VDZ, in patients with CD (Table 1; Figure 5A and Figure S6) and in patients with UC (Table 2; Figure 5B and Figure S7). 

## 11. Discussion

This is the first systematic review to comprehensively compare the efficacy and safety of maintenance treatment with IFX versus VDZ in patients with moderately to severely active CD and moderately to severely active UC based on state-of-the-art trial data and including results from the LIBERTY studies [9]. The results show that maintenance treatment with IFX yielded statistically significantly better effectiveness than VDZ in patients with CD, in terms of both clinical remission (CDAI score ≤ 150) and clinical response (CDAI-100). In patients with UC, IFX and VDZ yielded similar rates of clinical remission and clinical response (based on the total/partial Mayo score), and similar rates of mucosal healing and CS-free remission. In both populations (CD and UC), the proportions of patients experiencing AEs, SAEs, serious infections, or discontinuation due to AEs did not differ significantly between IFX and VDZ. Additionally, sensitivity analyses were conducted for the primary and co-primary efficacy outcomes in patients with CD or UC who received IFX SC (pooled data were not available for VDZ SC) and in VDZ-treated, TNFi-naïve patients (i.e., excluding TNFi-experienced patients). The findings of both sensitivity analyses were consistent with those of the main analyses.

Notably, a more marked treatment difference (IFX vs. VDZ) was observed for the primary and co-primary efficacy endpoints in the CD population compared with the UC population. Pharmacokinetic or pharmacodynamic differences between agents, or differences in the expression of inflammatory cytokines during CD and UC pathogenesis, may potentially account for this finding. For example, CD pathogenesis is more commonly associated with TNF-driven immunopathology than UC [33,34]. As IFX is a direct inhibitor of TNF, this might account for the improved performance of IFX versus VDZ in CD but not in UC. Recent evidence has also shown that SC IFX is equally effective across all parts of the intestines, unlike most other drugs currently used to treat CD that typically show lower efficacy in patients with ileal involvement [35].

The findings of this study in patients with CD are consistent with those of a recent network meta-analysis of efficacy, which reported IFX (in particular, IFX SC) as ranking higher for the achievement of clinical remission in TNFi-naïve patients with CD compared with the VDZ IV/SC regimens evaluated [15]. Importantly, the present study extends these findings both through the evaluation of multiple efficacy outcomes and safety outcomes, and through the inclusion of data from the recent large-scale LIBERTY studies. In terms of efficacy, our findings differ slightly from those of an indirect comparison of data from IFX and VDZ randomized controlled trials in adults with moderate-to-severe IBD, which showed that IFX versus VDZ had comparable efficacy in the maintenance phase [3], noting that the indirect comparison did not include data from the LIBERTY studies. A recent comparative analysis of three CD and four UC randomized controlled trials (not including the LIBERTY studies) reported numerically, but not significantly, higher proportions of patients achieving clinical response and clinical remission with IFX SC versus VDZ [4]. An additional exploratory analysis was performed upon publication of the LIBERTY studies; the findings did not change with respect to patients with CD, and clinical responses and remissions remained similar with IFX SC versus VDZ in patients with UC [4]. These results differ from our findings of significantly better efficacy with maintenance IFX SC versus VDZ.

Findings from broader network meta-analyses are conflicting [12,13]. A network meta-analysis of TNFis, anti-integrin, anti–interleukin-12/23p40, and anti–interleukin-23p19 agents found that no individual agent was clearly superior for the maintenance of clinical remission in patients with CD [13]. However, a second network meta-analysis, which included the evaluation of specific regimens of biological therapies and small molecules for the maintenance of clinical remission in patients with luminal CD, showed that upadacitinib 30 mg once daily was superior to VDZ IV 300 mg Q4W/Q8W, VDZ SC 108 mg Q2W, and IFX IV 5 mg/kg Q8W, but not to IFX IV 10 mg/kg Q8W or IFX SC 120/240 mg Q2W [12].

Participant demographics and disease characteristics were representative of patients with moderately to severely active IBD, adding to the generalizability of these findings. While the included studies evaluating IFX enrolled almost exclusively TNFi-naïve populations (only the NOR-SWITCH study enrolled a small proportion [~5%] of biologic-experienced patients), TNFi-experienced patients were enrolled in several of the VDZ studies. To address this potential confounder, we conducted a sensitivity analysis to examine the effect of excluding VDZ-treated, TNFi-experienced patients. While pooled results for the primary and co-primary efficacy outcomes in VDZ-treated, TNFi-naïve patients with CD and UC were numerically higher than those reported in the main analyses (i.e., including VDZ-treated, TNFi-experienced patients), the trend was generally consistent when comparing with IFX in the main analyses.

In terms of the quality of the evidence, studies contributing data to the quantitative analyses were generally considered to have a low risk for selection bias, detection bias, attrition bias, and reporting bias. One study was judged to be at high risk of performance bias due to an open-label design, and the majority of studies were considered to be at high risk of other bias.

The levels of heterogeneity observed in several of the meta-analyses are potentially suggestive of inter-trial variation for IFX and VDZ, which is possibly related to differences in study design (i.e., the re-randomization of induction responders) or differences in endpoint definitions and prior treatment histories. As summarized above, our sensitivity analysis did not identify a substantial effect for prior TNFi exposure on the VDZ analyses, and the IFX studies almost exclusively enrolled TNFi-naïve individuals. Similarly, findings of a second sensitivity analysis in IFX SC-treated individuals were consistent with the main IFX analyses (i.e., including both IV- and SC-treated patients). Together, these findings suggest that differences in prior TNFi exposure or in IFX formulation are unlikely to account for the observed heterogeneity across VDZ and IFX studies, respectively.

While the current study showed no significant differences between IFX and VDZ with regard to safety, and is aligned with a previous systematic review and meta-analysis in which proportions of patients reporting serious infections did not differ between IFX and VDZ [3], it should be appreciated that this is not a universal finding [36]. However, any differences that have been reported when treating IBD do not consistently favor either agent across the entire safety profile [36].

The present study has several strengths and limitations. Strengths include the use of robust, validated methodology (e.g., comprehensive electronic searches, independent screening of search results by two authors, assessment of risk of bias using an established tool) [37], and the inclusion of the most recent data for IFX SC from the LIBERTY randomized controlled trials [9] and for VDZ SC from the VISIBLE 2 study [10]. Potential limitations include that the LIBERTY trials randomized patients for maintenance at week 10, following the US regimen (maintenance dosage starting after three IV administrations), while other trials, such as the VISIBLE and GEMINI trials, randomized patients at week 6. Additionally, the analyzed population included a mixture of biologic-experienced and biologic-naïve individuals; many VDZ-treated patients were TNFi experienced, whereas most IFX-treated patients were TNFi naïve. However, the findings of the sensitivity analysis to exclude TNFi-experienced patients were consistent with those of the main analyses. We acknowledge the limitations of indirect comparisons, including differences in study design, populations, and interventions, which may introduce heterogeneity. For instance, several outcomes showed high heterogeneity, reducing the power of the pooled results; many trials randomized only responders, potentially introducing bias; and most trials were sponsored by pharmaceutical companies, raising the potential risk of publication bias. Additionally, factors such as disease location (e.g., ileal vs. colonic Crohn’s disease), the presence of immune markers, or anti-drug antibodies may affect drug efficacy; however, the analysis was not stratified by these factors. Furthermore, SC and IV formulations have different absorption and blood concentrations, which were not adjusted for in the analysis. Additionally, we were unable to examine treatment effects solely in trials involving treat-through study designs in patients with CD or UC, or treatment effects solely in trials involving re-randomization of responders in patients with UC alone, as there was an insufficient number of trials to permit comparison (i.e., at least two trials per treatment are needed).

Finally, given the limited duration of follow-up (i.e., the one-year timeframe inherent to all the included studies), important longer-term differences in efficacy or safety might not be detected. There was limited power to detect any differences between the treatments with regards to safety, or to detect rarer safety events occurring with longer exposure. Long-term data from extension studies were not considered in the present study, as extension studies are standardly open-label and often lack a comparator group.

## 12. Future Perspectives

Future research should incorporate an analysis of drug costs and patient quality of life, as these factors are important considerations in therapy selection.

## 13. Conclusions

An indirect comparison of maintenance treatment with IFX and VDZ, using data from randomized control trials, demonstrated that IFX yields significantly better efficacy in terms of clinical remission and clinical response than VDZ in patients with CD, while IFX and VDZ yield similar efficacy in patients with UC, including similar rates of clinical remission, clinical response, mucosal healing, and CS-free remission. Safety outcomes were either similar or numerically better with IFX than VDZ in patients with CD or UC.

## Figures and Tables

**Table 1:** Summary of findings—Crohn’s disease.

Outcome	Maintenance Treatment	Estimate (95% CI)	Heterogeneity, *I^2^*
Clinical remission (CDAI score ≤ 150)	IFX	0.64 (0.60–0.68)	16%
	VDZ	0.40 (0.35–0.46)	65%
Clinical response (CDAI-100)	IFX	0.71 (0.67–0.75)	25%
	VDZ	0.47 (0.43–0.51)	32%
Mucosal healing	IFX	0.31 (0.26–0.36)	22%
	VDZ	–	–
CS-free remission	IFX	0.40 (0.34–0.47)	55%
	VDZ	0.34 (0.27–0.41)	58%
Any AEs	IFX	0.75 (0.66–0.84)	91%
	VDZ	0.83 (0.77–0.89)	90%
Any SAEs	IFX	0.12 (0.08–0.16)	76%
	VDZ	0.18 (0.12–0.24)	91%
Any serious infection	IFX	0.03 (0.02–0.05)	0%
	VDZ	0.04 (0.02–0.06)	74%
Discontinuation due to AEs	IFX	0.07 (0.01–0.13)	90%
	VDZ	0.09 (0.06–0.12)	79%

AE, adverse event; CDAI, Crohn’s Disease Activity Index; CI, confidence interval; CS, corticosteroid; IFX, infliximab; SAE, serious adverse event; VDZ, vedolizumab.

**Table 2:** Summary of findings—ulcerative colitis.

Outcome	Maintenance Treatment	Estimate (95% CI)	Heterogeneity, *I^2^*
Clinical remission (total/partial Mayo score) ^a^	IFX	0.54 (0.38–0.71)	96%
	VDZ	0.40 (0.35–0.44)	67%
Clinical response (total/partial Mayo score) ^b^	IFX	0.52 (0.45–0.58)	55%
	VDZ	0.58 (0.51–0.65)	68%
Mucosal healing	IFX	0.48 (0.41–0.54)	77%
	VDZ	0.48 (0.39–0.57)	92%
CS-free remission	IFX	0.34 (0.27–0.40)	0%
	VDZ	0.28 (0.19–0.38)	88%
Any AEs	IFX	0.74 (0.64–0.83)	91%
	VDZ	0.76 (0.69–0.83)	92%
Any SAEs	IFX	0.12 (0.07–0.17)	80%
	VDZ	0.11 (0.08–0.13)	38%
Any serious infection	IFX	0.03 (0.02–0.04)	22%
	VDZ	0.02 (0.01–0.02)	0%
Discontinuation due to AEs	IFX	0.04 (0.02–0.06)	61%
	VDZ	0.06 (0.03–0.09)	62%

^a^ A total/partial Mayo score ≤ 2 with no individual sub-score > 1; or a partial Mayo score ≤1 with a rectal bleeding sub-score of 0; or a modified Mayo score with a stool frequency sub-score of ≤1, rectal bleeding sub-score of 0, and endoscopic sub-score of ≤1. ^b^ A decrease in the total Mayo score from a baseline of ≥3 points and ≥30% (or a decrease in the partial Mayo score from a baseline of ≥2 points and ≥25%), with an accompanying decrease in the rectal bleeding sub-score of ≥1 or an absolute rectal bleeding sub-score of ≤1. AE, adverse event; CI, confidence interval; CS, corticosteroid; IFX, infliximab; SAE, serious adverse event; VDZ, vedolizumab.

## Data Availability

The original contributions presented in this study are included in the article/Supplementary Materials.

## Supplementary Materials

The following supporting information can be downloaded at: https://www.mdpi.com/article/10.3390/jcm14134419/s1, Figure S1. Risk of bias for the included studies, presented by study and domain (A) and by domain only (B). Figure S2. Sensitivity analysis 1. Pooled rates of clinical remission by CDAI (A), clinical response by CDAI-100 (B), clinical remission by total/partial Mayo score (C), and clinical response by total/partial Mayo score (D) during maintenance treatment with IFX in patients with CD (A, B) and UC (C, D). Figure S3. Sensitivity analysis 2. Pooled rates of clinical remission by CDAI, clinical response by CDAI-100, and CS-free remission during maintenance treatment with VDZ in TNFi-naïve (A–C) or TNFi-experienced patients with CD (D–F). Figure S4. Sensitivity analysis 2. Pooled rates of clinical remission by total/partial Mayo score (A), mucosal healing (B) and CS-free remission (C) during maintenance treatment with VDZ in TNFi-naïve patients with UC. Figure S5. Sensitivity analysis 3. Pooled rates of clinical remission by CDAI (A) and clinical response by CDAI-100 (B) during maintenance treatment with IFX or VDZ in patients with CD. Figure S6. Forest plots showing the proportion of participants with CD who experienced any adverse event (A), serious adverse event (B), serious infection (C), and/or adverse events leading to discontinuation (D) during maintenance treatment with IFX (A) or VDZ (B). Figure S7. Forest plots showing the proportion of participants with UC who experienced any adverse event (A), serious adverse event (B), serious infection (C), and/or adverse events leading to discontinuation (D) during maintenance treatment with IFX (A) or VDZ (B). Table S1. Core search terms. Table S2. Characteristics of the included studies (CD). Table S3. Characteristics of the included studies (UC).
