# Supplementary material for: Efficacy and Safety of Infliximab and Vedolizumab Maintenance Therapy in Patients with Crohn’s Disease and Ulcerative Colitis: A Systematic Review and Meta-Analysis
Source: J Clin Med. 2025 Jun 21;14(13):4419. doi: 10.3390/jcm14134419 (PMC12249907; doi:10.3390/jcm14134419)
Supplement: Supplementary file 1 [file jcm-14-04419-s001.zip › jcm-3662334-supplementary.pdf]

Supplementary Tables

Table S1. Core search terms.

Topic	Query
Participants	"crohn*"[Title/Abstract] OR "CD"[Title/Abstract] OR "ulcerative colitis"[Title/Abstract] OR "ulcerative"[Title/Abstract] OR "UC"[Title/Abstract] OR "IBD"[Title/Abstract] OR "inflammatory bowel disease"[Title/Abstract] OR "Crohn Disease"[MeSH Terms] OR "colitis, ulcerative"[MeSH Terms]
Intervention	"subcutaneous infliximab"[Title] OR "infliximab subcutaneous"[Title] OR "SC infliximab"[Title] OR "infliximab SC"[Title] OR "IFX subcutaneous"[Title] OR "subcutaneous IFX"[Title] OR "IFX SC"[Title] OR "SC IFX"[Title] OR "CT-P13"[Title] OR "infliximab"[Title] OR "remicade"[Title] OR "remsima"[Title] OR "inflectra"[Title] OR "infliximab#dyyb"[Title] OR "CTP13"[Title] OR "CT#P13"[Title] OR "Infliximab#abda"[Title] OR "Renflexis"[Title] OR "Flixabi"[Title] OR "SB2"[Title] OR "SB#2"[Title] OR "infliximab#qbtx"[Title] OR "Ixifi"[Title] OR "Zessly"[Title] OR "PF#06438179"[Title] OR "PF06438179"[Title] OR "PF#6438179"[Title] OR "PF6438179"[Title] OR "GP1111"[Title] OR "GP#1111"[Title] OR "infliximab#axxq"[Title] OR "Avsola"[Title] OR "ABP710"[Title] OR "ABP#710"
Comparator	"vedolizumab"[Title] OR "Entyvio"[Title] OR "MLN0002"[Title] OR "MLN02"[Title] OR "LDP-02"[Title]
Study design	"randomized controlled trial"[Publication Type] OR "controlled clinical trial"[Publication Type] OR ("randomised"[Title/Abstract] OR "randomized"[Title/Abstract]) OR "placebo"[Title/Abstract] OR "randomly"[Title/Abstract] OR "trial"[Title/Abstract] NOT "animals"[MeSH Terms] NOT "humans"[MeSH Terms]

CD, Crohn’s disease; IBD, inflammatory bowel disease; MeSH, Medical Subject Headings; SC, subcutaneous; UC, ulcerative colitis.

Table S2. Characteristics of the included studies (CD).

Study name	Study duration, weeks	N, total enrollment	Maintenance regimen, N patients	Age, median (range), years	Sex, male, %	Disease duration, mean (SD), years	BMI, median (range), kg/m²	CDAI score, mean (SD)	Fecal calprotectin, mean (SD), mg/kg	SES-CD, mean (SD)	Prior TNFi exposure, n (%)
**IFX**											
CT-P13 1.6 [23]	54	Induction: 136<br>Maintenance: 131[a]	CT-P13 SC 120/240 mg[b] Q2W: CD, 28<br>CT-P13 IV 5 mg/kg Q8W: CD, 25	34.0 (18–69)<br>35.0 (19–53)	57.1<br>44.0	4.5 (6.6)<br>5.6 (5.6)	23.4 (16.4–34.7)<br>22.8 (15.3–33.7)	296.4 (59.2)<br>294.8 (59.9)	3,045.0 (5,966.3)<br>4,027.0 (13,736.5)	10.9 (8.3)<br>8.1 (5.8)	0<br>0
LIBERTY-CD [9]	54	Induction: 396<br>Maintenance: 343	CT-P13 SC 120 mg Q2W: 231<br>Placebo: 112	36.0 (25–43)<br>29.0 (23–39)	58.0<br>61.6	4.3 (5.2)[c]<br>4.5 (5.8)[c]	22.7 (14.2–34.6)<br>21.8 (15.9–34.7)	312.0 (58.4)<br>309.7 (62.6)	2,585.2 (7,612.4)<br>1,951.6 (2,554.9)	11.5 (6.9)<br>11.7 (6.2)	0<br>0
NOR-SWITCH [20, 21]	52	Maintenance: 248[d]	CT-P13 IV 5 mg/kg Q8W: CD, 77<br>IFX IV 5 mg/kg Q8W: CD, 78	39.5 (14.2)[e]<br>38.0 (13.4)[e]	61.0<br>57.7	14.3 (8.5)<br>12.8 (9.0)	NR<br>NR	NR<br>NR	65 (27–210)[f]<br>70 (32–191)[f]	NR<br>NR	17 (22.1)<br>17 (21.8)
PLANET-CD [22]	54	Randomized: 220	CT-P13 IV → CT-P13 IV (5 mg/kg Q8W): 56[g]<br>CT-P13 IV → IFX (5 mg/kg Q8W): 55[g]<br>IFX → IFX (5 mg/kg Q8W): 54[g]<br>IFX → CT-P13 IV (5 mg/kg Q8W): 55[g]	39.0 (19.0–68.0)<br>31.0 (18.0–69.0)<br>31.0 (18.0–69.0)<br>35.0 (19.0–63.0)	51.8<br>61.8<br>48.1<br>61.8	NR<br>NR<br>NR<br>NR	NR<br>NR<br>NR<br>NR	291.0 (52.7)<br>301.8 (55.9)<br>292.9 (57.4)<br>298.5 (53.9)	516.5 (10–9,932)[h]<br>532.0 (10–8,360)[h]<br>488.0 (10–11,673)[h]<br>449.0 (10–11,389)[h]	10.3 (9.1)<br>10.4 (7.8)<br>11.7 (8.6)<br>7.8 (6.7)	0<br>0<br>0<br>0
SONIC [19]	50	Randomized: 508	IFX IV 5 mg/kg Q8W: 97[i]<br>IFX IV 5 mg/kg Q8W + AZA: 108[i]<br>Placebo + AZA QD: 75[i]	35 (NR)<br>34 (NR)<br>35 (NR)	49.7<br>52.1<br>52.9	2.2 (NR)[h]<br>2.2 (NR)[h]<br>2.4 (NR)[h]	NR<br>NR<br>NR	284.8 (62.1)<br>289.9 (55.0)<br>287.2 (52.9)	NR<br>NR<br>NR	NR<br>NR<br>NR	0<br>0<br>0
**VDZ**											
GEMINI 2 [24, 25]	52	Induction: 1,115<br>Maintenance: 461	VDZ IV 300 mg Q8W: 154<br>VDZ IV 300 mg Q4W: 154<br>Placebo: 153<br>Week 6 non-responders VDZ IV 300 mg Q4W: 506	35.1 (12.2)[e]<br>34.9 (12.2)[e]<br>37.3 (12.0)[e]<br>35.8 (11.7)[e]	44.2<br>53.2<br>47.1<br>45.3	8.4 (7.3)<br>7.7 (6.8)<br>9.6 (8.9)<br>9.7 (7.8)	NR<br>NR<br>NR<br>NR	326 (69)<br>317 (66)<br>325 (66)<br>324 (69)	584 (NR)[h]<br>776 (NR)[h]<br>684 (NR)[h]<br>702 (NR)[h]	NR<br>NR<br>NR<br>NR	88 (57)<br>83 (54)<br>82 (54)<br>364 (72)
VISIBLE 2 [10]	52	Induction: 644<br>Maintenance: 410	VDZ SC 108 mg Q2W: 275<br>Placebo: 134	38.2 (13.9)[e]<br>36.1 (12.9)[e]	57.1<br>49.3	9.5 (8.3)<br>8.2 (8.4)	NR<br>NR	318.8 (206.0–559.0)[i]<br>309.0 (198.0–461.0)[i]	736.0 (10–14,570)[h]<br>870.5 (10–15,050)[h]	NR<br>NR	168 (61.1)<br>71 (53.0)

[a]CD, n=53; UC, n=78.

[b]Body weight-based dosing: 120 mg Q2W for <80 kg and 240 mg Q2W for ≥80 kg.

[c]Time since active CD diagnosis.

[d]CD, n=155; UC, n=93.

[e]Data are mean (SD).

[f]Data are median (25–75 percentiles).

[g]Maintenance with six doses of study drug Q8W from Week 14 to Week 54, with patients switching to second treatment at Week 30.

[h]Data are median (range).

[i]Patients who completed 30-week trial and enrolled in 20-week extension.

AZA, azathioprine; BMI, body mass index; CD, Crohn's disease; CDAI, Crohn's Disease Activity Index, IFX, infliximab; IV, intravenous; NR, not reported; Q*n*W, every *n* weeks; SC, subcutaneous; SD, standard deviation; SES-CD, Simple Endoscopic Score for Crohn's Disease; TNFi, tumor necrosis factor inhibitor; UC, ulcerative colitis; VDZ, vedolizumab.

Table S3. Characteristics of the included studies (UC).

Study name	Study duration, weeks	N, total enrollment	Maintenance regimen, N patients	Age, median (range), years	Sex, male, %	Disease duration, mean (SD), years	BMI, median (range), kg/m²	Mayo score, mean (SD)	Fecal calprotectin, mean (SD), mg/kg	Prior TNFi exposure, n (%)
**IFX**										
ACT 1 [26, 27]	54	Randomized: 364	IFX IV 5 mg/kg Q8W: 121<br>IFX IV 10 mg/kg Q8W: 122<br>Placebo: 121	42.4 (14.3)[a]<br>41.8 (14.9)[a]<br>41.4 (13.7)[a]	64.5<br>59.0<br>59.5	5.9 (5.4)<br>8.4 (8.1)<br>6.2 (5.9)	NR<br>NR<br>NR	8.5 (1.7)<br>8.4 (1.4)<br>8.4 (1.8)	NR<br>NR<br>NR	0<br>0<br>0
CT-P13 1.6 [23]	54	Induction: 136<br>Maintenance: 131[c]	CT-P13 SC 120/240 mg[d] Q2W: UC, 38<br>CT-P13 IV 5 mg/kg Q8W: UC, 40	33.0 (18–65)<br>37.0 (18–70)	52.6<br>60.0	6.6 (5.5)<br>6.0 (6.7)	23.9 (18.4–34.9)<br>24.1 (15.6–38.3)	7.9 (1.6)<br>8.2 (1.7)	1,393.7 (1,603.5)<br>1,693.7 (2,408.6)	0<br>0
LIBERTY-UC [9]	54	Induction: 538<br>Maintenance: 438	CT-P13 SC 120 mg Q2W: 294<br>Placebo: 144	37 (29–47)<br>39 (30–50)	55.4<br>57.6	6.1 (6.0)[e]<br>6.8 (6.8)[e]	23.3 (15.1–36.0)<br>24.7 (16.5–34.5)	8.8 (1.3)<br>8.8 (1.4)	2,681.0 (3,467.6)<br>2,441.2 (3,142.9)	0<br>0
NOR-SWITCH [20, 21]	52	Maintenance: 248[f]	CT-P13 IV 5 mg/kg Q8W: UC, 46<br>IFX IV 5 mg/kg Q8W: UC, 47	44.4 (14.8)[a]<br>45.8 (14.1)[a]	69.6<br>61.7	11.5 (7.5)<br>11.2 (9.2)	NR<br>NR	0 (0–1)[g,h]<br>0 (0–1)[g,h]	39.5 (19–208)[g]<br>44 (19–111)[g]	3 (7.0)<br>2 (4.3)
**VDZ**										
GEMINI 1 [28, 29]	52	Induction: 895<br>Maintenance: 373	VDZ IV 300 mg Q8W: 122<br>VDZ IV 300 mg Q4W: 125<br>Placebo: 126<br>Week 6 non-responders VDZ IV 300 mg Q4W: 373	41.0 (13)[a]<br>38.6 (14)[a]<br>40.3 (14)[a]<br>40.3 (13)[a]	57.4<br>54.4<br>54.8<br>60.6	6.2 (5)<br>7.6 (7)<br>7.8 (7)<br>6.5 (6)	NR<br>NR<br>NR<br>NR	8.4 (1.8)<br>8.3 (1.7)<br>8.4 (1.8)<br>8.7 (1.8)	864 (281–1,727)[g]<br>793 (332–1,654)[g]<br>1,071 (331–2,553)[g]<br>852 (385–1,790)[g]	50 (41)<br>52 (42)<br>47 (37)<br>209 (56)
VARSITY [30]	52	Randomized: 769	VDZ IV 300 mg Q8W + placebo SC Q2W: 385<br>ADA 40 mg Q2W weeks + placebo IV Q8W: 386	40.8 (13.7)[a]<br>40.5 (13.4)[a]	60.8<br>56.0	7.3 (7.2)<br>6.4 (6.0)	NR<br>NR	8.7 (1.6)<br>8.7 (1.5)	2,929 (5,920)<br>2,771 (4,064)	152 (39.5)<br>160 (41.5)
VISIBLE 1 [11]	52	Induction: 383<br>Maintenance: 216	VDZ SC 108 mg Q2W + placebo IV Q8W: 106<br>VDZ IV 300 mg Q8W + placebo SC Q2W: 54<br>Placebo SC Q2W + placebo IV Q8W: 56	38.1 (13.1)[a]<br>41.6 (14.1)[a]<br>39.4 (11.7)[a]	61.3<br>57.4<br>60.7	8.0 (6.2)<br>8.2 (5.9)<br>7.4 (7.1)	NR<br>NR<br>NR	9.0 (6–12)[i]<br>9.0 (6–12)[i]<br>9.0 (6–11)[i]	1,735 (42–15,696)[i]<br>1,589 (130–28,490)[i]<br>1,554 (30–13,620)[i]	40 (37.7)<br>24 (44.4)<br>20 (35.7)

[a]Data are mean (SD).

[b]Body weight, kg.

[c]CD, n=53; UC, n=78.

[d]Body weight-based dosing: 120 mg Q2W for <80 kg and 240 mg Q2W for ≥80 kg.

[e]Time since active CD diagnosis.

[f]CD, n=155; UC, n=93.

[g]Data are median (25–75 percentiles).

[h]Partial Mayo score.

[i]Data are median (range).

ADA, adalimumab; BMI, body mass index; CD, Crohn's disease; IV, intravenous; Q*n*W, every *n* weeks; SC, subcutaneous; SD, standard deviation; TNFi, tumor necrosis factor inhibitor; UC, ulcerative colitis; VDZ, vedolizumab.

Figure S1. Risk of bias for the included studies, presented by study and domain (A) and by domain only (B).

A

	Random sequence generation (selection bias)	Allocation concealment (selection bias)	Blinding of participants & personnel (performance bias)	Blinding of outcome assessment (detection bias)	Incomplete outcome data (attrition bias)	Selective reporting (reporting bias)	Other bias
ACT1	+	+	+	+	+	+	+
GEMINI1	+	+	+	+	+	+	−
GEMINI2	+	+	+	+	+	+	−
LIBERTY-CD	+	+	+	+	+	+	−
LIBERTY-UC	+	+	+	+	+	+	−
NCT02883452	+	+	−	+	+	+	+
NOR-SWITCH	+	+	+	+	+	+	+
PLANET-CD	+	+	+	+	+	+	−
SONIC	+	+	+	+	+	+	+
VARSITY	+	+	+	+	+	+	+
VISIBLE1	+	+	+	+	+	+	−
VISIBLE2	+	+	+	+	+	+	−

B

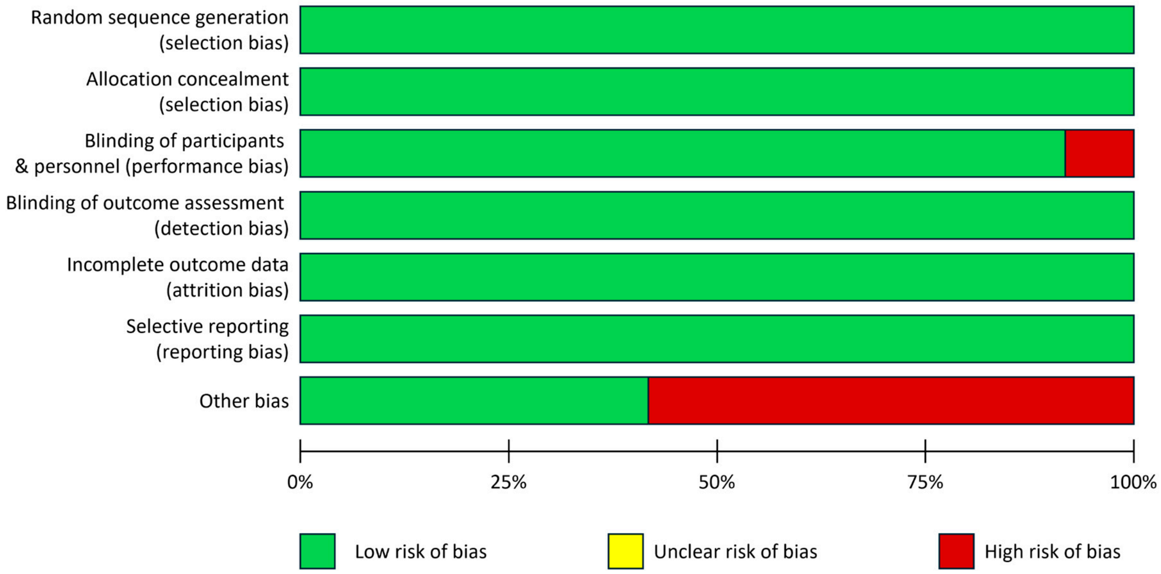
Random sequence generation
(selection bias)
Allocation concealment
(selection bias)
Blinding of participants
& personnel (performance bias)
Blinding of outcome assessment
(detection bias)
Incomplete outcome data
(attrition bias)
Selective reporting
(reporting bias)
Other bias
0%
25%
50%
75%
100%
Low risk of bias
Unclear risk of bias
High risk of bias

Figure S2. Sensitivity analysis 1. Pooled rates of clinical remission by CDAI (A), clinical response by CDAI-100 (B), clinical remission by total/partial Mayo score (C), and clinical response by total/partial Mayo score (D) during maintenance treatment with IFX in patients with CD (A, B) and UC (C, D).

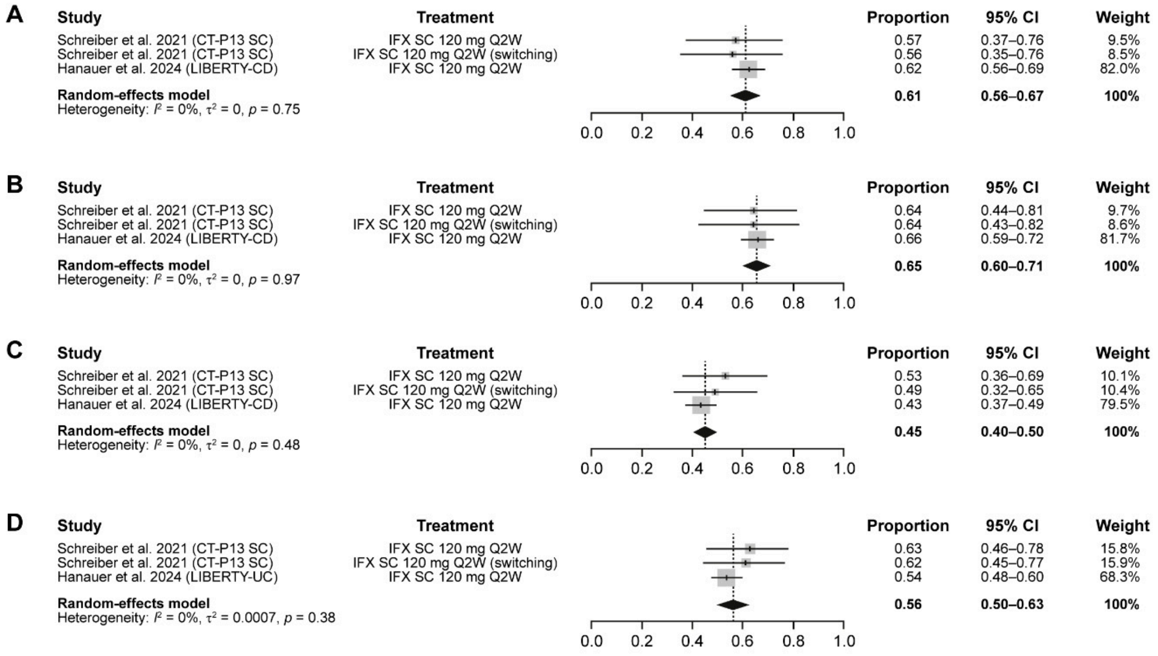

CD, Crohn's disease; CDAI, Crohn's Disease Activity Index; CI, confidence interval; IFX, infliximab; Q2W, every 2 weeks; SC, subcutaneous; UC, ulcerative colitis.

Figure S3. Sensitivity analysis 2. Pooled rates of clinical remission by CDAI, clinical response by CDAI-100, and CS-free remission during maintenance treatment with VDZ in TNFi-naïve (A–C) or TNFi-experienced patients with CD (D–F).

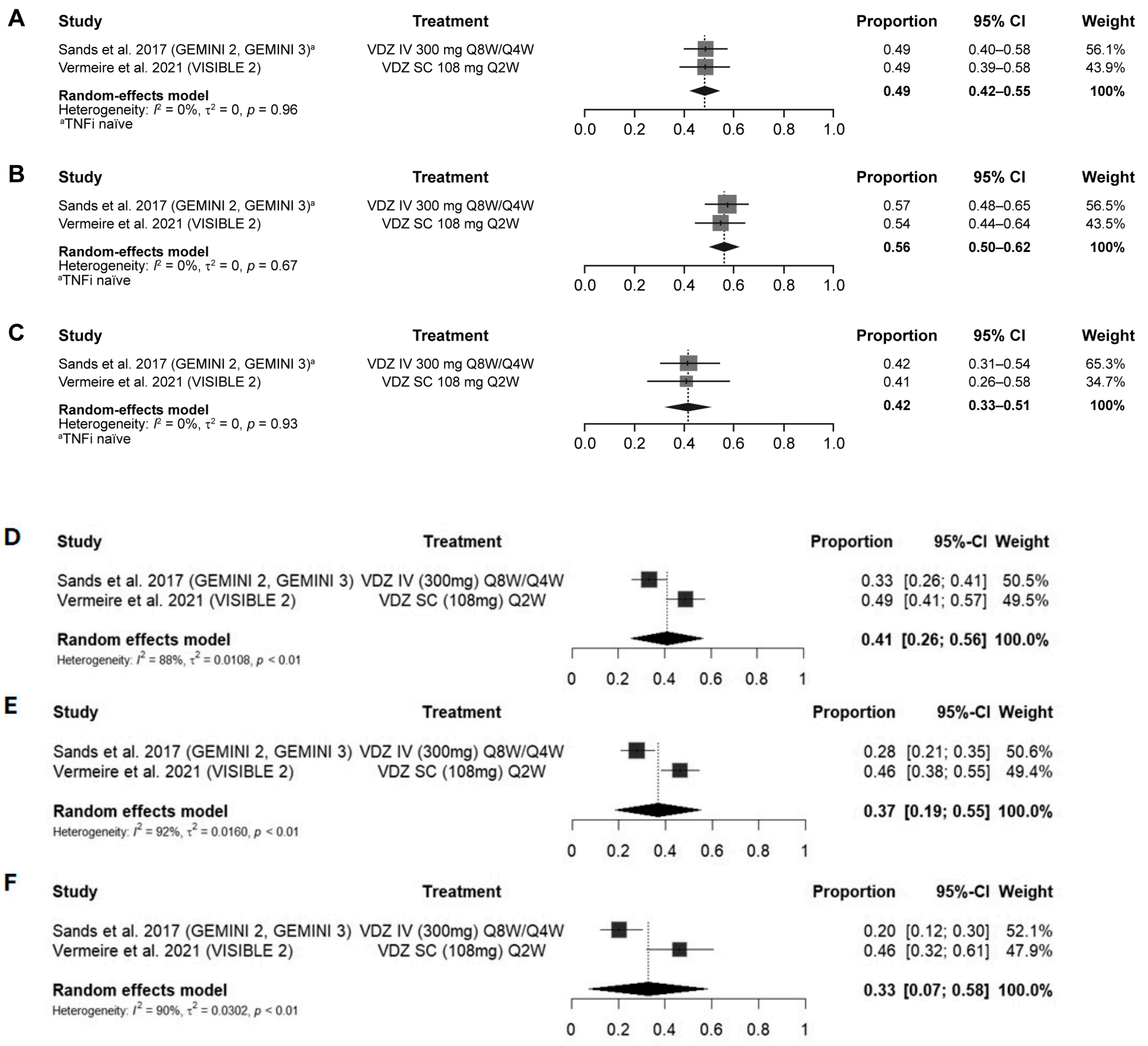

[a]TNF naïve.

[b]TNF failure.

CD, Crohn's disease; CDAI, Crohn's Disease Activity Index; CI, confidence interval; CS, corticosteroid; IV, intravenous; Q*n*W, every *n* weeks; SC, subcutaneous; TNFi, tumor necrosis factor inhibitor; VDZ, vedolizumab.

Figure S4. Sensitivity analysis 2. Pooled rates of clinical remission by total/partial Mayo score (A), mucosal healing (B) and CS-free remission (C) during maintenance treatment with VDZ in TNFi-naïve patients with UC.

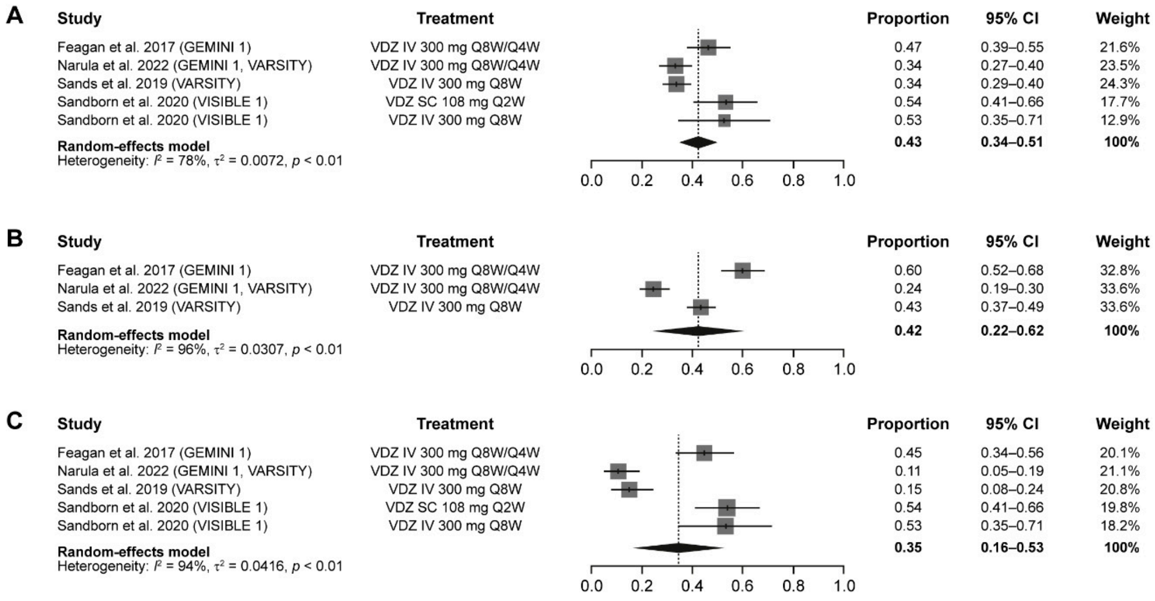

CI, confidence interval; CS, corticosteroid; IV, intravenous; Q*n*W, every *n* weeks; SC, subcutaneous; TNFi, tumor necrosis factor inhibitor; UC, ulcerative colitis; VDZ, vedolizumab.

Figure S5. Sensitivity analysis 3. Pooled rates of clinical remission by CDAI (A) and clinical response by CDAI-100 (B) during maintenance treatment with IFX or VDZ in patients with CD.

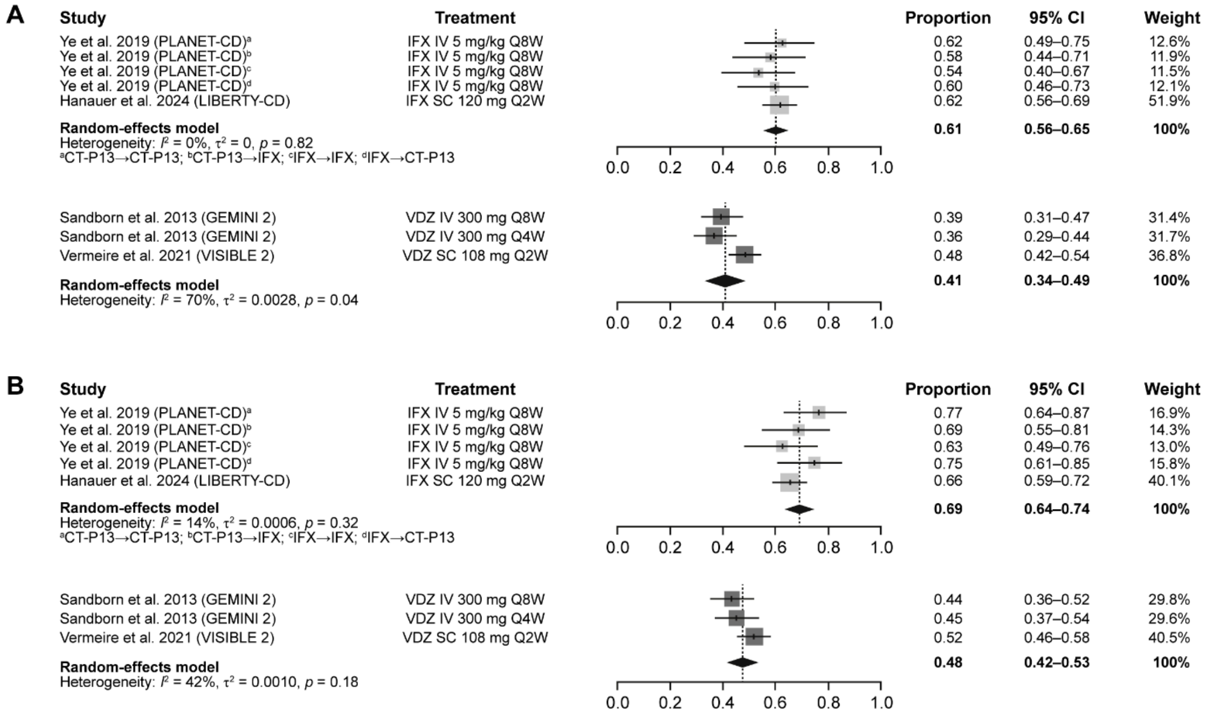

Note: Due to rounding, some totals are not 100%.

CD, Crohn's disease; CDAI, Crohn's Disease Activity Index; CI, confidence interval; IFX, infliximab; IV, intravenous; Q*n*W, every *n* weeks; SC, subcutaneous; VDZ, vedolizumab.

Figure S6. Forest plots showing the proportion of participants with CD who experienced any adverse event (A), serious adverse event (B), serious infection (C), and/or adverse events leading to discontinuation (D) during maintenance treatment with IFX (A) or VDZ (B).

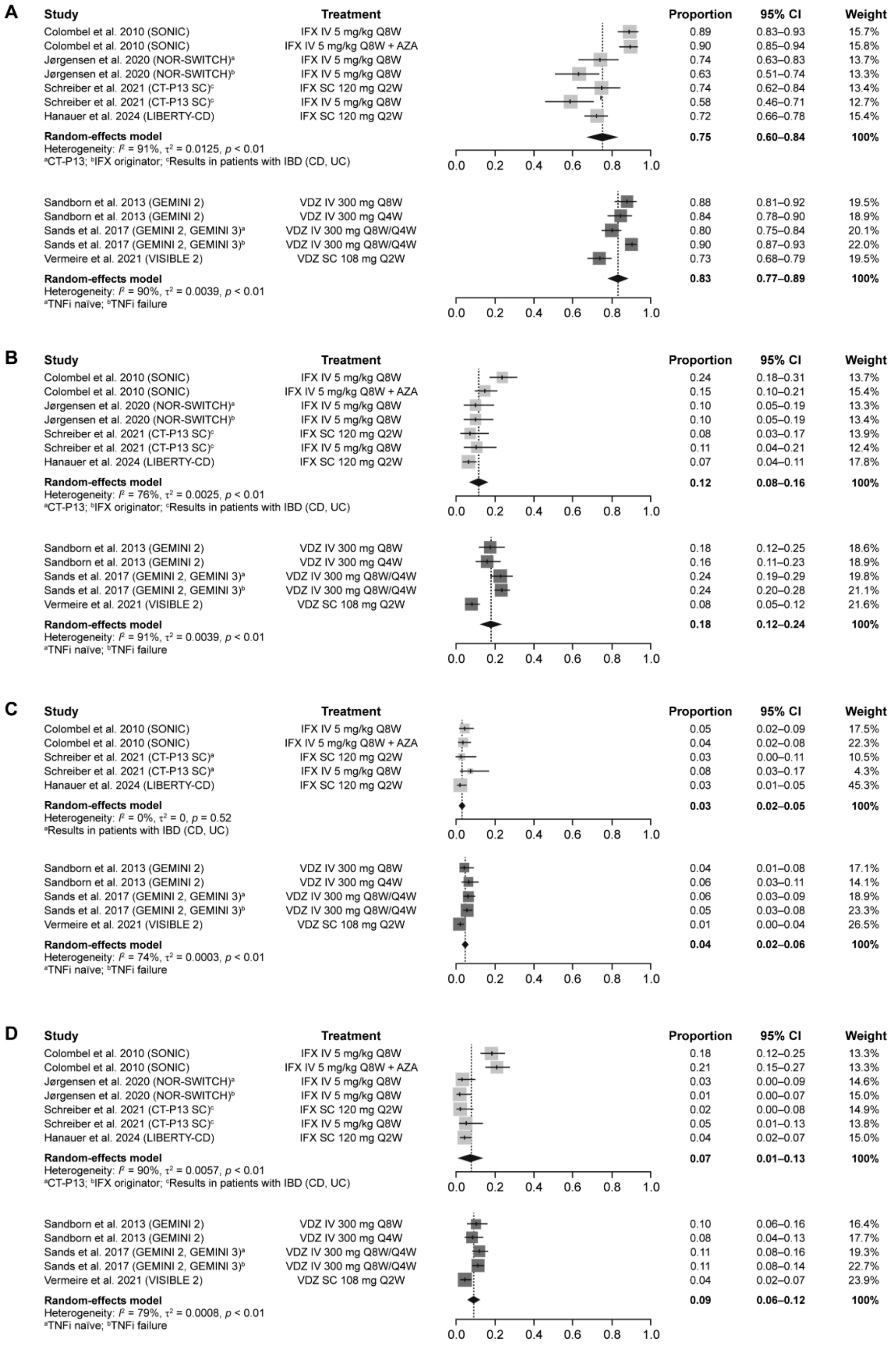

Note: Due to rounding, some totals are not 100%.

AZA, azathioprine; CD, Crohn’s disease; CI, confidence interval; IBD, inflammatory bowel disease; IFX, infliximab; IV, intravenous; Q*n*W, every *n* weeks; SC, subcutaneous; TNFi, tumor necrosis factor inhibitor; UC, ulcerative colitis; VDZ, vedolizumab.

Figure S7. Forest plots showing the proportion of participants with UC who experienced any adverse event (A), serious adverse event (B), serious infection (C), and/or adverse events leading to discontinuation (D) during maintenance treatment with IFX (A) or VDZ (B).

A

Study	Treatment	Proportion	95% CI	Weight
Rutgeerts et al. 2005 (ACT 1)	IFX IV 5 mg/kg Q8W	0.88	0.80–0.93	15.8%
Rutgeerts et al. 2005 (ACT 1)	IFX IV 10 mg/kg Q8W	0.91	0.84–0.95	16.1%
Jørgensen et al. 2020 (NOR-SWITCH)[a]	IFX IV 5 mg/kg Q8W	0.63	0.48–0.77	12.3%
Jørgensen et al. 2020 (NOR-SWITCH)[b]	IFX IV 5 mg/kg Q8W	0.68	0.53–0.81	12.6%
Schreiber et al. 2021 (CT-P13 SC)[c]	IFX SC 120 mg Q2W	0.74	0.62–0.84	13.9%
Schreiber et al. 2021 (CT-P13 SC)[c]	IFX IV 5 mg/kg Q8W	0.58	0.46–0.71	13.2%
Hanauer et al. 2024 (LIBERTY-UC)	IFX SC 120 mg Q2W	0.68	0.62–0.73	16.0%
**Random-effects model**		**0.74**	**0.64–0.83**	**100%**

Heterogeneity: $I^2 = 91\%$, $\tau^2 = 0.0136$, $p < 0.01$
[a]CT-P13; [b]IFX originator; [c]Results in patients with IBD (CD, UC)

Study	Treatment	Proportion	95% CI	Weight
Feagan et al. 2013 (GEMINI 1)	VDZ IV 300 mg Q8W	0.82	0.74–0.88	14.4%
Feagan et al. 2013 (GEMINI 1)	VDZ IV 300 mg Q4W	0.81	0.73–0.87	14.3%
Feagan et al. 2017 (GEMINI 1)[a]	VDZ IV 300 mg Q8W/Q4W	0.74	0.69–0.79	15.4%
Feagan et al. 2017 (GEMINI 1)[b]	VDZ IV 300 mg Q8W/Q4W	0.88	0.83–0.91	15.8%
Sands et al. 2019 (VARSITY)	VDZ IV 300 mg Q8W	0.63	0.58–0.68	15.4%
Sandborn et al. 2020 (VISIBLE 1)	VDZ SC 108 mg Q2W	0.65	0.55–0.74	13.0%
Sandborn et al. 2020 (VISIBLE 1)	VDZ IV 300 mg Q8W	0.76	0.62–0.87	11.6%
**Random-effects model**		**0.76**	**0.69–0.83**	**100%**

Heterogeneity: $I^2 = 92\%$, $\tau^2 = 0.0077$, $p < 0.01$
[a]TNFi naïve; [b]TNFi failure

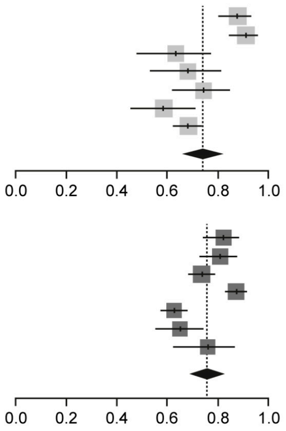

B

Study	Treatment	Proportion	95% CI	Weight
Rutgeerts et al. 2005 (ACT 1)	IFX IV 5 mg/kg Q8W	0.21	0.15–0.30	13.7%
Rutgeerts et al. 2005 (ACT 1)	IFX IV 10 mg/kg Q8W	0.24	0.17–0.32	13.5%
Jørgensen et al. 2020 (NOR-SWITCH)[a]	IFX IV 5 mg/kg Q8W	0.07	0.01–0.18	13.9%
Jørgensen et al. 2020 (NOR-SWITCH)[b]	IFX IV 5 mg/kg Q8W	0.09	0.02–0.20	13.1%
Schreiber et al. 2021 (CT-P13 SC)[c]	IFX SC 120 mg Q2W	0.08	0.03–0.17	14.6%
Schreiber et al. 2021 (CT-P13 SC)[c]	IFX IV 5 mg/kg Q8W	0.11	0.04–0.21	13.5%
Hanauer et al. 2024 (LIBERTY-UC)	IFX SC 120 mg Q2W	0.06	0.04–0.10	17.6%
**Random-effects model**		**0.12**	**0.07–0.17**	**100%**

Heterogeneity: $I^2 = 80\%$, $\tau^2 = 0.0040$, $p < 0.01$
[a]CT-P13; [b]IFX originator; [c]Results in patients with IBD (CD, UC)

Study	Treatment	Proportion	95% CI	Weight
Feagan et al. 2013 (GEMINI 1)	VDZ IV 300 mg Q8W	0.08	0.04–0.15	13.4%
Feagan et al. 2013 (GEMINI 1)	VDZ IV 300 mg Q4W	0.09	0.04–0.15	13.0%
Feagan et al. 2017 (GEMINI 1)[a]	VDZ IV 300 mg Q8W/Q4W	0.09	0.06–0.13	21.0%
Feagan et al. 2017 (GEMINI 1)[b]	VDZ IV 300 mg Q8W/Q4W	0.17	0.12–0.22	14.9%
Sands et al. 2019 (VARSITY)	VDZ IV 300 mg Q8W	0.11	0.08–0.15	21.4%
Sandborn et al. 2020 (VISIBLE 1)	VDZ SC 108 mg Q2W	0.09	0.05–0.17	11.2%
Sandborn et al. 2020 (VISIBLE 1)	VDZ IV 300 mg Q8W	0.13	0.05–0.25	5.3%
**Random-effects model**		**0.11**	**0.08–0.13**	**100%**

Heterogeneity: $I^2 = 38\%$, $\tau^2 = 0.0003$, $p = 0.14$
[a]TNFi naïve; [b]TNFi failure

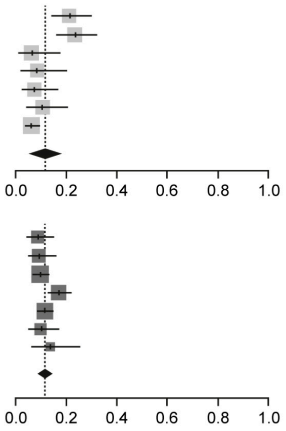

C

Study	Treatment	Proportion	95% CI	Weight
Rutgeerts et al. 2005 (ACT 1)	IFX IV 5 mg/kg Q8W	0.02	0.01–0.07	22.9%
Rutgeerts et al. 2005 (ACT 1)	IFX IV 10 mg/kg Q8W	0.07	0.03–0.13	9.5%
Schreiber et al. 2021 (CT-P13 SC)[a]	IFX SC 120 mg Q2W	0.03	0.00–0.11	10.7%
Schreiber et al. 2021 (CT-P13 SC)[a]	IFX IV 5 mg/kg Q8W	0.08	0.03–0.17	4.4%
Hanauer et al. 2024 (LIBERTY-UC)	IFX SC 120 mg Q2W	0.02	0.01–0.05	52.4%
**Random-effects model**		**0.03**	**0.02–0.04**	**100%**

Heterogeneity: $I^2 = 22\%$, $\tau^2 < 0.0001$, $p = 0.27$
[a]Results in patients with IBD (CD, UC)

Study	Treatment	Proportion	95% CI	Weight
Feagan et al. 2013 (GEMINI 1)	VDZ IV 300 mg Q8W	0.02	0.01–0.07	6.4%
Feagan et al. 2013 (GEMINI 1)	VDZ IV 300 mg Q4W	0.02	0.00–0.06	10.0%
Feagan et al. 2017 (GEMINI 1)[a]	VDZ IV 300 mg Q8W/Q4W	0.01	0.00–0.03	30.4%
Feagan et al. 2017 (GEMINI 1)[b]	VDZ IV 300 mg Q8W/Q4W	0.03	0.01–0.06	11.5%
Sands et al. 2019 (VARSITY)	VDZ IV 300 mg Q8W	0.02	0.01–0.04	26.8%
Sandborn et al. 2020 (VISIBLE 1)	VDZ SC 108 mg Q2W	0.02	0.00–0.07	7.2%
Sandborn et al. 2020 (VISIBLE 1)	VDZ IV 300 mg Q8W	0.00	0.00–0.07	7.7%
**Random-effects model**		**0.02**	**0.01–0.02**	**100%**

Heterogeneity: $I^2 = 0\%$, $\tau^2 = 0$, $p = 0.67$
[a]TNFi naïve; [b]TNFi failure

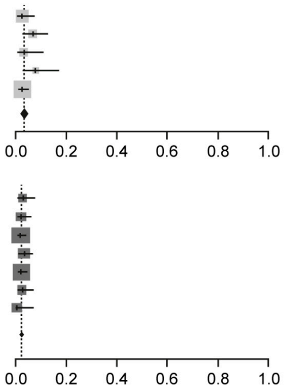

D

Study	Treatment	Proportion	95% CI	Weight
Rutgeerts et al. 2005 (ACT 1)	IFX IV 5 mg/kg Q8W	0.08	0.04–0.15	11.9%
Rutgeerts et al. 2005 (ACT 1)	IFX IV 10 mg/kg Q8W	0.09	0.05–0.16	11.5%
Jørgensen et al. 2020 (NOR-SWITCH)[a]	IFX IV 5 mg/kg Q8W	0.00	0.00–0.08	17.5%
Jørgensen et al. 2020 (NOR-SWITCH)[b]	IFX IV 5 mg/kg Q8W	0.04	0.01–0.15	10.0%
Schreiber et al. 2021 (CT-P13 SC)[c]	IFX SC 120 mg Q2W	0.02	0.00–0.08	17.5%
Schreiber et al. 2021 (CT-P13 SC)[c]	IFX IV 5 mg/kg Q8W	0.05	0.01–0.13	11.5%
Hanauer et al. 2024 (LIBERTY-UC)	IFX SC 120 mg Q2W	0.03	0.02–0.06	20.1%
**Random-effects model**		**0.04**	**0.02–0.06**	**100%**

Heterogeneity: $I^2 = 61\%$, $\tau^2 = 0.0006$, $p = 0.02$
[a]CT-P13; [b]IFX originator; [c]Results in patients with IBD (CD, UC)

Study	Treatment	Proportion	95% CI	Weight
Feagan et al. 2013 (GEMINI 1)	VDZ IV 300 mg Q8W	0.06	0.02–0.11	19.4%
Feagan et al. 2013 (GEMINI 1)	VDZ IV 300 mg Q4W	0.05	0.02–0.10	21.0%
Sands et al. 2019 (VARSITY)	VDZ IV 300 mg Q8W	0.11	0.08–0.15	23.7%
Sandborn et al. 2020 (VISIBLE 1)	VDZ SC 108 mg Q2W	0.05	0.02–0.11	19.8%
Sandborn et al. 2020 (VISIBLE 1)	VDZ IV 300 mg Q8W	0.04	0.00–0.13	16.1%
**Random-effects model**		**0.06**	**0.03–0.09**	**100%**

Heterogeneity: $I^2 = 62\%$, $\tau^2 = 0.0006$, $p = 0.03$

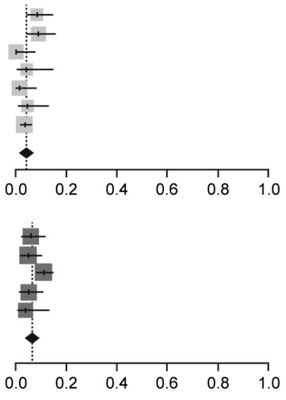

Note: Due to rounding, some totals are not 100%.

CD, Crohn's disease; CI, confidence interval; IBD, inflammatory bowel disease; IFX, infliximab; IV, intravenous; Q*n*W, every *n* weeks; SC, subcutaneous; TNFi, tumor necrosis factor inhibitor; UC, ulcerative colitis; VDZ, vedolizumab.
